# Serological analysis of Ebola virus survivors and close contacts in Sierra Leone: A cross-sectional study

**DOI:** 10.1371/journal.pntd.0007654

**Published:** 2019-08-01

**Authors:** Peter J. Halfmann, Amie J. Eisfeld, Tokiko Watanabe, Tadashi Maemura, Makoto Yamashita, Satoshi Fukuyama, Tammy Armbrust, Isaiah Rozich, Alhaji N’jai, Gabriele Neumann, Yoshihiro Kawaoka, Foday Sahr

**Affiliations:** 1 Department of Pathobiological Sciences, School of Veterinary Medicine, University of Wisconsin, Madison, Wisconsin, United States of America; 2 Institute of Medical Science, University of Tokyo, Tokyo, Japan; 3 Department of Biological Sciences, Fourah Bay College, University of Sierra Leone, Freetown, Sierra Leone; 4 34^th^ Regimental Military Hospital at Wilberforce, Freetown, Sierra Leone; Institute for Disease Modeling, UNITED STATES

## Abstract

The 2013–2016 Ebola virus outbreak in West Africa was the largest and deadliest outbreak to date. Here we conducted a serological study to examine the antibody levels in survivors and the seroconversion in close contacts who took care of Ebola-infected individuals, but did not develop symptoms of Ebola virus disease. In March 2017, we collected blood samples from 481 individuals in Makeni, Sierra Leone: 214 survivors and 267 close contacts. Using commercial, quantitative ELISAs, we tested the plasma for IgG-specific antibodies against three major viral antigens: GP, the only viral glycoprotein expressed on the virus surface; NP, the most abundant viral protein; and VP40, a major structural protein of *Zaire ebolavirus*. We also determined neutralizing antibody titers. In the cohort of Ebola survivors, 97.7% of samples (209/214) had measurable antibody levels against GP, NP, and/or VP40. Of these positive samples, all but one had measurable neutralizing antibody titers against Ebola virus. For the close contacts, up to 12.7% (34/267) may have experienced a subclinical virus infection as indicated by detectable antibodies against GP. Further investigation is warranted to determine whether these close contacts truly experienced subclinical infections and whether these asymptomatic infections played a role in the dynamics of transmission.

## Author summary

As the causative agent of an often lethal hemorrhagic fever disease in humans and nonhuman primates, *Zaire ebolavirus* typically causes high fever, severe diarrhea, and vomiting which results in case fatality rates as high as 90%. The 2013–2016 outbreak in West Africa was the largest and most devastating Ebola outbreak to date resulting in over 28,600 identified human cases and 11,300 deaths. Though our knowledge of virus transmission is incomplete, we do know that transmission occurs through direct contact with virus-contaminated body fluids (blood, secretions, or other body fluids), materials such as bedding contaminated with these fluids, and through the handling and preparation of contaminated food. Asymptomatic Ebola virus infections that result in seroconversion in the absence of disease symptoms have been observed both in humans and experimentally in animal models. In the present serology study, we determined a majority of Ebola survivors in our cohort had measurable antibody levels against at least one viral antigen, as expected. In our cohort of close contacts, relatives and health care workers who took care of Ebola-infected individuals during the outbreak, we observed a rate of seroprevalence of 12.7% as indicated by detectable GP antibody levels. Given that Ebola virus is typically associated with a highly lethal disease in humans, it is of great interest to determine the host-virus interactions and transmission dynamics associated with asymptomatic cases.

## Introduction

There are six antigenically distinct species in the genus Ebolavirus that vary in viral pathogenesis. Infections caused by *Zaire ebolavirus* result in the highest lethality in humans with case fatality rates during outbreaks ranging from 41% to 90% (average rate, 78%). Ebola virus (EBOV) is typically introduced into human populations through direct contact with or the consumption of infected nonhuman primates or other intermediate mammalian hosts or through bats, a potential natural reservoir of EBOV [1]. Human-to-human transmission occurs through direct contact with virus-laden secretions or fluids [2]. Initial symptoms of EBOV infection include fever, cough, rash, and abdominal pain, which occur within 2 to 21 days of contact with the virus, and progress to fatigue, headache, vomiting, diarrhea, shock, organ failure, and potential death.

A total of 14 documented EBOV outbreaks have been reported in Central Africa. The 2013–2016 EBOV outbreak in West Africa was the first for this region of Africa; it was also the largest and most devastating EBOV outbreak to date resulting in over 28,600 identified human cases and 11,300 deaths. These figures include 881 cases of infected health care workers, including 513 deaths. The outbreak was located primarily in the West African countries of Sierra Leone, Liberia, and Guinea, but seven other countries experienced imported cases.

Although the highly pathogenic nature of EBOV is well-established, several studies have assessed the incidence of asymptomatic infections that result in seroconversion in the absence of symptoms of disease [3–11]. These studies report a wide variability of seroprevalence, ranging from 1.0% to 45.9%, which precludes an accurate summary estimate of asymptomatic human cases. In addition to human cases, asymptomatic cases have been documented experimentally in animal models such as ferrets [12] and nonhuman primates [13].

Limited information is available regarding the antibody status of survivors of the West African outbreak and the number of asymptomatic cases that occurred in Sierra Leone. To address this lack of information, we obtained samples from EBOV survivors and from individuals who cared for virus-infected patients either at home or in treatment centers. We assessed antibody levels in these samples by using an ELISA against the three major viral antigens, GP, NP, and VP40; we also evaluated neutralizing antibody titers.

## Methods

### Study site, questionnaire, and blood sample collection

The study was carried out in Makeni (estimated population of 112,428 in 2013), the capital of the Bombali District of Sierra Leone, which experienced 1,050 confirmed EBOV cases during the 2014–2016 outbreak. Recruitment of adult volunteers (survivors and close contacts) was performed by the Sierra Leone Association of Ebola Survivors of Makeni. Demographic data and information were collected using a questionnaire. The study also included a control cohort of 38 individuals with no known exposure to EBOV and no relationship to EBOV-infected individuals.

A peripheral blood sample (~3 ml) was collected in an EDTA vacutainer tube (Becton Dickinson, Franklin Lakes, NJ) by local, experienced technicians at the Makeni medical center. Blood samples were stored at 4°C for less than 24 hours prior to isolation of the plasma fraction. The plasma was then treated at 55°C for 30 minutes, divided into aliquots, and stored for use at -80°C.

### Serologic assays

By using commercial, quantitative ELISAs (Alpha Diagnostics International, San Antonio, Texas), we tested the plasma obtained from the blood samples at a 1:400 dilution at least in duplicate for levels of IgG-specific antibodies against the three major viral antigens: the surface GP, the only virally expressed protein on the virion surface; NP, the most abundant viral protein; and VP40, a major structural protein of EBOV (strain Mayinga). Using the calibrators provided in the ELISA kits, a threshold index for each ELISA run was established to discriminate between positive and negative antibody responses and determine antibody levels (expressed in units/ml). Distribution of antibody levels and determination of mean antibody levels were determined using GraphPad Prism 7.

Neutralizing antibody titers were determined using our replication-defective EBOVΔVP30 system that lacks the essential VP30 protein, but undergoes efficient replication in cell lines that are genetically engineered to stably express VP30 [14]. The virus system is approved for biosafety level-2 containment at the University of Wisconsin and is excluded from the CDC’s Select Agent registration. Serial 2-fold dilutions (1:4 to 1:1,024) of heat-inactivated plasma samples were mixed with an equal volume of ~1,000 focus-forming units of EBOVΔVP30 containing the Renilla luciferase reporter gene that resulted in an additional 2-fold dilution of the plasma sample. The virus-antibody mixture (in duplicate) was used to infect 96-well plates of VeroVP30 cells, a Vero cell lines that express the EBOV VP30 gene in order to facilitate EBOVΔVP30 replication. Three days after infection, a live-cell luciferase reagent was added to the wells, and luciferase activity (a measurement of virus replication) was determined as relative light units (RLU). Neutralizing antibody titers were defined as the highest plasma dilution that resulted in a 50% reduction in RLU compared to a plasma control.

### Ethical aspects

The study was approved by the Ethical Review Board of the Ministry of Health and Sanitation of Sierra Leone and the Human Subjects Institutional Review Boards at the University of Wisconsin and the University of Tokyo. All participants in this study were adults. Inclusion in the study was voluntary with written informed consent provided by obtaining a signature or fingerprint from each participant and a signature from a witness.

## Results

In March 2017, a total of 481 blood samples were collected from a cohort in Makeni, Sierra Leone, consisting of survivors of the 2013–2016 EBOV outbreak (n = 214) and individuals who had close contact with EBOV-infected individuals during their illness, but did not develop symptoms of Ebola virus disease (n = 267). In the cohort of close contacts, health care workers (n = 56) reported working at an Ebola treatment unit for a time period of 1–2 years and consistently used personal protective equipment during their interactions with EBOV-infected individuals. Four health care workers took part in an Ebola vaccine clinical trial and were not included in this study. Also in the cohort of close contacts were relatives (n = 211) who took care of a sick family member on average for 10 days before the infected family member was taken to a treatment unit. Only 66% of relatives reported using personal protective equipment while taking care of sick family members due to the limited access to these items.

**Fig 1:**
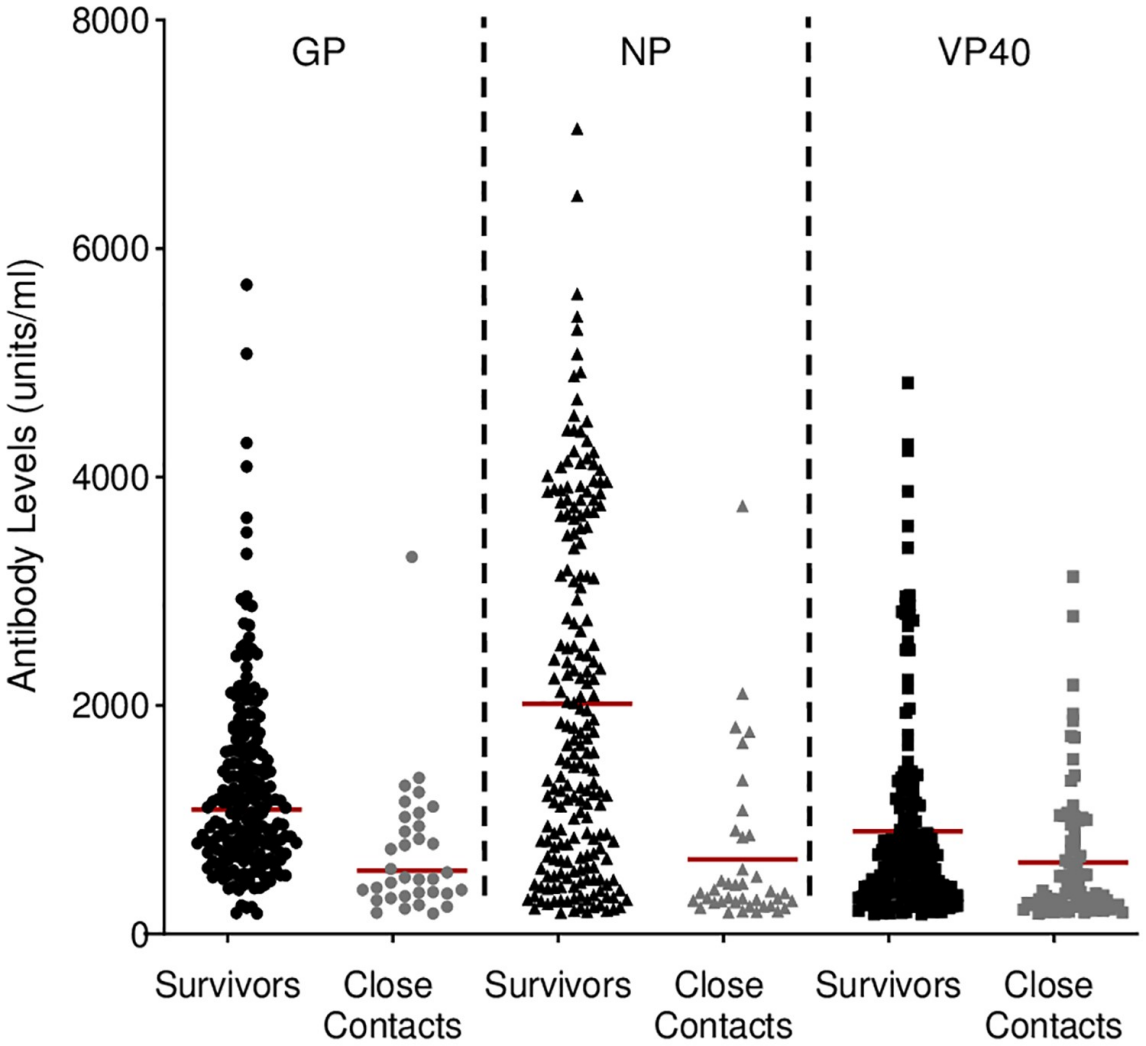
IgG-specific antibody levels (units/ml) against three viral antigens as determined by quantitative ELISA using plasma obtained from survivors and close contacts. The red bar indicates the geometric mean.

The blood samples were collected from survivors 15–32 months (median = 28 months) after recovery from infection and release from a treatment unit. In this cohort, 85.0%, 10.7%, and 1.9% of samples were positive for antibodies against all three, two, or one viral antigen by ELISA, respectively, while 2.3% of the samples were negative against all three antigens (Table 1). In the survivor cohort, the mean NP antibody level was 2,015 units/ml, which was higher than the mean antibody levels against the other two antigens (GP and VP40) (Fig 1 and Table 2).

**Table 1:** Summary of serology among EBOV survivors and close contacts against different viral antigens (GP, NP, and/or VP40)*.

	Antibodies against:				
	**3 antigens**	**2 antigens**	**1 antigen**	**0 antigens**	**TOTAL**
Number of Survivors (percentage)	182 (85.0%)	23 (10.7%)	4 (1.9%)	5 (2.3%)	214 (97.7% positive ≥ one antigen)
Number of Survivors with antibodies against specific antigens		12 –GP and NP 11 –GP and VP40 0 –NP and VP40	4 –GP 0 –NP 0 –VP40		
	**3 antigens**	**2 antigens**	**1 antigen**	**0 antigens**	**TOTAL**
Number of Close Contacts^#^ (percentage)	18 (6.7%) 0 HCWs 18 relatives	20 (7.5%) 3 HCWs 17 relatives	69 (25.8%) 13 HCWs 56 relatives	160 (59.9%) 40 HCWs 120 relatives	267 (40.1% positive ≥ one antigen) 56 HCWs 211 relatives
Number of Close Contacts with antibodies against specific antigens	18 –GP, NP, VP40	3 –GP and NP 6 –GP and VP40 11 –NP and VP40	7 –GP 8 –NP 54 –VP40		34 –GP (12.7%)

*A total of 481 blood samples were collected from a cohort in Makeni, Sierra Leone in March 2017.

^#^Close contacts are separated into two subgroups; health care workers (HCWs) and relatives. Samples were tested by quantitative ELISA for antibodies to GP, NP, and/or VP40.

**Table 2:** The range and mean of antibody levels against each viral antigen*.

Antigen	GP			NP			VP40		
	Min.	Max.	Mean	Min.	Max.	Mean	Min.	Max.	Mean
Survivors (n = 214)	179	5,685	1,300	186	7,051	2,015	175	4,825	899
Close contacts (n = 267)	180	3,302	701	188	3,748	652	178	3,128	627

* Minimum (Min.), maximum (Max.) and mean antibody levels are expressed as units/ml as determined by use of quantitative ELISAs.

In the cohort of close contacts, 6.7% of samples were positive for antibodies against all three viral antigens (0 health care workers and 18 relatives), 7.5% were positive for antibodies against two viral antigens (3 health care workers and 17 relatives), and 25.8% were positive for antibodies against one viral antigen (13 health care workers and 56 relatives) (Table 1). Of the close contacts who were positive for antibodies against one or two antigens, a majority of samples (n = 71) possessed antibodies against VP40. In general, the mean antibody levels against each viral antigen measured in the close contact cohort were lower than those in the survivor cohort (Fig 1 and Table 2). As a comparison, we examined antibodies against GP in a limited control cohort from Makeni (n = 38), but we were unable to detect measurable antibody levels (S1 Table).

**Fig 2:**
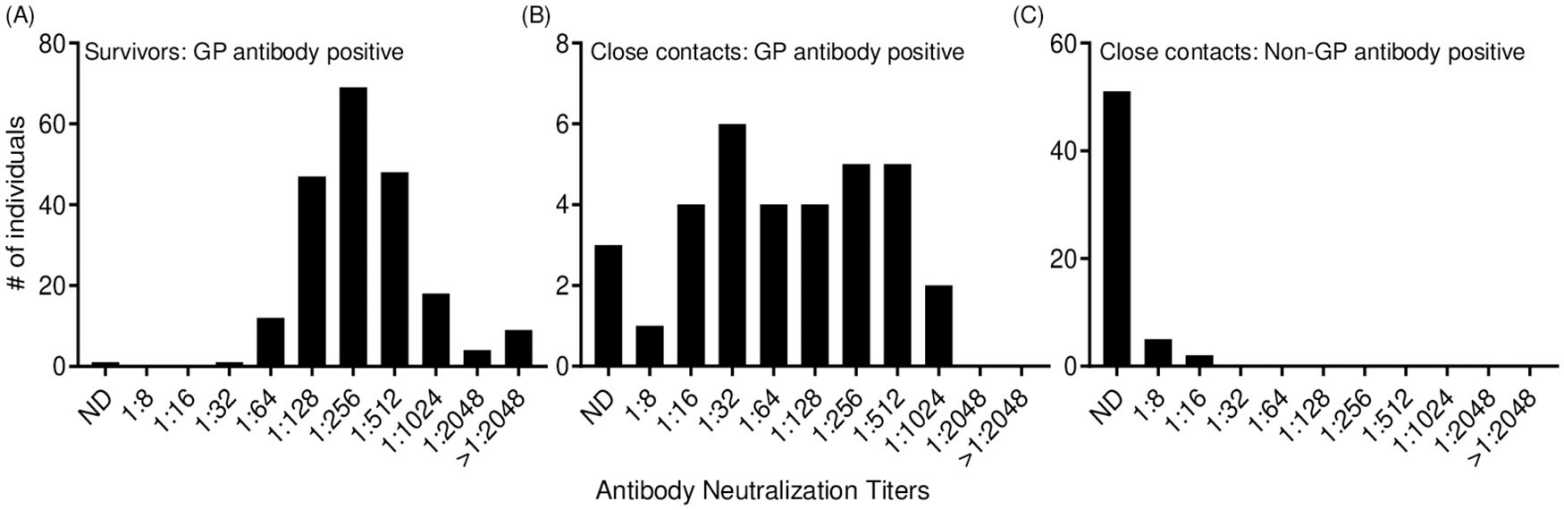
Distribution of individuals with detectable neutralizing antibody titers. (A) Survivors who were positive for GP antibodies. (B) Close contacts who were positive for GP antibodies. (C) Close contact who were positive for non-GP antibodies (NP and/or VP40). Note the differences in the scale of the Y-axis in each graph.

Next, we examined the samples for neutralizing antibodies. Survivors that were positive for GP antibodies by ELISA (n = 209) also had detectable neutralizing antibody titers (except for one survivor) with the majority of survivors having titers ranging from 1:128 to 1:512, but some survivors had titers of great than 1:2048 (Fig 2A). Close contacts that were positive for GP antibodies by ELISA (n = 34) had a range of neutralizing antibody titers from 1:8 to 1:1024 while 3 of these samples had no detectable neutralizing titer (Fig 2B). For close contacts that were antibody positive for other viral antigens (NP and/or VP40, but not GP; n = 73), the majority of these close contact samples had an undetectable neutralizing antibody titer; however, titers were detected in 7 individuals at 1:8 and 1:16 (Fig 2C).

## Discussion

While one research group has examined the T-cell response in EBOV survivors of the West Africa outbreak [15], little is known about the antibody status of survivors of the West Africa outbreak. Here, we demonstrate that two years after infection, 97.7% of EBOV survivors in our study have measurable antibody levels against GP, NP, and/or VP40, and all of the survivors, but one had measurable neutralizing antibody titers against EBOV. The lack of detectable antibody levels in some survivors (2.3%, n = 5) could reflect immune defects resulting in low and/or short-lived antibody responses, or could be due to technical errors or miscommunication during sample collection.

Serology studies have examined the incidence of asymptomatic EBOV cases in populations living in endemic and non-endemic areas as well as in populations that have known and unknown contacts with infected individuals (reviewed in [4]). In these studies, EBOV-specific antibody levels were assessed by using different techniques (e.g., immunofluorescence assay and commercial or ‘home-made’ ELISA kits), which most likely contributed to the wide variation in the seroprevalence rate of 1%–45.9%, depending on the method of antibody detection used [4]. In our study of asymptomatic cases, we determined antibody levels against three major viral antigens by using commercial ELISA kits and supplemented our findings by determining neutralizing antibody titers. Given protein homology of EBOV NP and VP40 to related viruses such as paramyxoviruses or rhabdoviruses, there could be cross-reactivity resulting in false-positive results in the NP and VP40 ELISAs [16]. Therefore, we based our incidence of asymptomatic infections on antibodies against GP such that 34 close contacts (12.7%) were positive for GP antibodies, and a majority of individuals (91.2%) also had measurable neutralizing antibody titers. However, it is unknown when or how these individuals were exposed to EBOV or if they were exposed to a filovirus antigenically similar to EBOV.

Asymptomatic cases have been documented for different viral infections including influenza virus and Zika virus [17–19]. For EBOV, these asymptomatic cases may be influenced by the route of infection, the exposure dose, or both. A recent nonhuman primate study demonstrated that a low challenge dose of EBOV (10 virus particles) by oral inoculation resulted in virus shedding, but never resulted in any clinical signs of infection [13]. Similar subclinical cases have been observed in the ferret model of EBOV infection. While infected ferrets developed clinical symptoms of EBOV infection, non-experimentally infected, cage mates that had direct contact with infected animals developed antibodies against EBOV, but never showed any signs of illness [12]. Given that our knowledge of EBOV transmission between individuals is incomplete, it is important to study these asymptomatic cases further to clarify their potential role in the transmission dynamics of an EBOV outbreak.

## Supporting information

S1 Table Raw GP ELISA data from a control cohort in Makeni, Sierra Leone. (XLSX)
